# Countering DDoS Attacks in SIP Based VoIP Networks Using Recurrent Neural Networks

**DOI:** 10.3390/s20205875

**Published:** 2020-10-17

**Authors:** Waleed Nazih, Yasser Hifny, Wail S. Elkilani, Habib Dhahri, Tamer Abdelkader

**Affiliations:** 1 College of Computer Engineering and Sciences, Prince Sattam Bin Abdulaziz University, Al Kharj 11942, Saudi Arabia; w.nazeeh@psau.edu.sa; 2 Faculty of Computers and Information Sciences, Ain Shams University, Abassia, Cairo 11566, Egypt; tammabde@cis.asu.edu.eg; 3 Faculty of Computers and Information, Helwan University, Ain Helwan, Cairo 11795, Egypt; yhifny@fci.helwan.edu.eg; 4 College of Applied Computer Sciences (CACS), King Saud University, Riyadh 11543, Saudi Arabia; hdhahri@ksu.edu.sa; 5 Faculty of Sciences and Technology, University of Kairouan, Sidi Bouzid 4352, Tunisia

**Keywords:** deep learning, recurrent neural networks, voice over IP, session initiation protocol, network security, distributed denial of service attacks

## Abstract

Many companies have transformed their telephone systems into Voice over IP (VoIP) systems. Although implementation is simple, VoIP is vulnerable to different types of attacks. The Session Initiation Protocol (SIP) is a widely used protocol for handling VoIP signaling functions. SIP is unprotected against attacks because it is a text-based protocol and lacks defense against the growing security threats. The Distributed Denial of Service (DDoS) attack is a harmful attack, because it drains resources, and prevents legitimate users from using the available services. In this paper, we formulate detection of DDoS attacks as a classification problem and propose an approach using token embedding to enhance extracted features from SIP messages. We discuss a deep learning model based on Recurrent Neural Networks (RNNs) developed to detect DDoS attacks with low and high-rate intensity. For validation, a balanced real traffic dataset was built containing three attack scenarios with different attack durations and intensities. Experiments show that the system has a high detection accuracy and low detection time. The detection accuracy was higher for low-rate attacks than that of traditional machine learning.

## 1. Introduction

Voice over IP (VoIP) is a collection of technologies and protocols used for transferring voice and multimedia over Internet Protocol (IP) networks. VoIP solutions are replacing traditional systems worldwide and are expected to be the dominant voice communications technology for fifth-generation (5G) networks. According to a recent report from Cisco [1], many organizations use VoIP services instead of their traditional telephone systems. The rapidly increasing number of VoIP users results in it being a target for attackers, which can decrease its Quality of Service (QoS) [2].

VoIP systems dependent on the underlying IP network infrastructure and use many protocols such as Real-Time Transport Protocol (RTP) [3] transferring voice and multimedia and Session Initiation Protocol (SIP) [4] for sessions communication. Consequently, VoIP systems inherited attacks that are generated from IP network protocols and vulnerable to attacks of its protocols [5]. VoIP-specific attacks are not detected by network security systems and therefore necessitate additional mechanisms for VoIP systems to identify and stop these types of attacks.

Recently, it appears that the intensity of attacks targeting VoIP networks have been growing [6], perhaps as a result of the rapid increase in the tools used by attackers and their capabilities. The full classification of VoIP attacks was explored in [7]. Any attack making a target SIP service or resource unavailable to legitimate users is a Denial of Service (DoS) attack. The attacker usually targets the SIP server to prevent subscribers from using VoIP services or to degrade the quality of offered services. DDoS attacks are always produced by using a group of computers controlled by the attacker (i.e., botnet), or by using one computer that generates malicious traffic as if it comes from multiple sources using IP spoofing.

DDoS are attacks considered one of the most dangerous attacks because they prevent legitimate users from using VoIP services. Moreover, these attacks can affect VoIP service availability by targeting one or many VoIP servers [8] thereby typically affecting working efficiency and possibly decreasing earnings.

Many researchers have used machine learning approaches to detect DDoS attacks as discussed in the next section. Those methods require comprehensive VoIP network knowledge to choose the proper features of SIP messages. In addition, thresholds and model parameters must be updated to be compatible with various types of DDoS attacks. Some of these approaches have not achieved high detection accuracy against low-rate DDoS attacks [9,10].

Deep learning is a form of machine learning that simulates the human brain using multi-layer neural networks, and large amounts of data must be used to train these neural networks’ parameters. One of the most valuable benefits of deep learning is its ability to learn features automatically and extract hidden relations through the use of many hidden layers. Substantial results have been achieved by deep learning in regard to speech recognition [11], speech synthesis [12], language translation [13], image classification [14], intrusion detection systems [15], and many other applications [16].

In this paper, we propose a detection approach that converts tokens of every SIP message into a feature vector and feeds those feature vectors into an RNN model to learn to detect DDoS attacks. Token embedding was used to enhance detection accuracy. Moreover, our approach processes SIP messages one by one and does not use a window size over SIP messages (e.g., 50 message) decreasing detection speed. Window sizes that were used in previously proposed approaches, such as the approach discussed in [9], are attack-dependent, thereby adding a limitation to these approaches in detecting different types of attacks. To our knowledge, our approach is the first that uses RNN to detect DDoS attacks in SIP-based VoIP networks. Our contributions include the following: (1) Introducing a novel DDoS detector using RNN; (2) Detecting low and high-rate DDoS attacks with high detection F1-score and low detection time over unseen dataset for the model (i.e., 99% and 0.16 ms per SIP message using RNN-GRU model); (3) Building a real balanced VoIP dataset to guarantee the reliability of the proposed approach; (4) Comparing our approach with classical machine learning approaches; (5) Testing the proposed approach on Graphics Processing Unit (GPU) and Central Processing Unit (CPU) systems.

The remainder of the paper is organized as follows. The next section summarizes related work. Section 3 describes the details of the proposed approach to the detection of DDoS attacks. Section 4 explains the dataset that we used for evaluation. Section 5 describes the experimental setup and results. Section 6 draws conclusions and discusses future work.

## 2. Related Work

Various approaches have been reported in the literature for detecting DDoS attacks in SIP-based VoIP networks. A real-time Packet-based SIP Intrusion Protector (PbSIP) has been proposed by Akbar et al. [17]. PbSIP is capable of detecting Spam over Internet Telephony (SPIT) attacks, DDoS flooding, and DoS flooding. PbSIP contains three modules, a packet analyzer module responsible for monitoring packets of SIP traffic, a feature extraction module for calculating features over a window of packets, and a Naive Bayes classifier for classifying messages as normal or malicious. PbSIP was tested over a set of different-intensity attack scenarios; it outperformed Support Vector Machines (SVMs) and Hellinger Distance (HD) in processing overhead and detection accuracy. Calculating features every 40 packets possibly delaying the raising of an attack alarm and low accuracy for low-rate attacks can be considered disadvantages of this approach.

Tag et al. [18] proposed a firewall component that uses a multi-dimensional sketch and HD. A three-dimensional sketch is used to convert SIP messages to a probability distribution over the sketch table. In addition, every SIP attribute (INVITE, OK, ACK, BYE) and its hash functions are stored. The HD value was used as an indicator of malicious traffic; a low HD value indicates that there is no change in SIP traffic, and a high value indicates there is SIP traffic deviation and that an anomaly has occurred. Although the proposed approach achieved promising results in DDoS attack detection, it failed to detect stealthy flooding attacks.

Tsiatsikas et al. [9] used machine learning techniques and SIP mandatory headers to detect DDoS attacks. They began by calculating the occurrences of six mandatory SIP headers over a window of SIP messages. Then, five classifiers were applied over various DoS and DDoS scenarios. To preserve user privacy, Hash-Based Message Authentication Code (HMAC) anonymization was used over SIP headers. The drawbacks of this approach are the low detection accuracy achieved in the case of low-rate attacks and the use of a large window of SIP messages, which can delay the raising of an attack alarm.

Tas et al. [19] proposed a two-module mechanism for protecting the SIP server from advanced DDoS attacks that take advantage of IP spoofing. The initial module is a statistics module that calculates dynamic thresholds over a specific SIP traffic duration. Then, a rules-based module uses those thresholds to take suitable action. The proposed mechanism lowered CPU processing of the SIP server under attack by 73.4%, but the detection accuracy against DDoS attacks was not reported.

Using the temporal features of the SIP state machine and a database of IP-fingerprints to detect and mitigate flooding attacks was proposed in [20]. At first, the state machine handles each SIP session and generates suitable events. Then, those events are fed into a detector to classify SIP sessions and store the session IP-fingerprint in the databases accordingly. Based on the decision of a filter module a message is allowed to pass or a mitigation process is started. The proposed approach was evaluated using different sites with attack sources, and real cloud scenarios for low and high-rate flooding and DDoS attacks. This reduced the computational resources used but with the use of many dynamic thresholds.

Semerci et al. [21] used a change-point model based on Mahalanobis distance for DDoS flooding attack detection and attacker identification. The proposed system monitors variation in Mahalanobis distance between successive feature vectors in a sampling interval (i.e., 1 to 10 s). If the Mahalanobis distance exceeds a pre-defined threshold, the system labels this as an attack. Furthermore, the system clusters the similarity scores of the users’ behavioral patterns to distinguish attackers from legitimate users. The proposed system used only the type and intensity of VoIP traffic but achieved a low accuracy (F1 score: 88%) using a ten second sampling interval, which is considered a long time for real-time systems to detect an attack. In addition, system parameters must be updated to account for VoIP traffic intensity.

Kurt et al. [10] extracted 41 features from SIP messages and resource usage measurements of the VoIP server to detect DDoS flooding attacks. Using a Hidden Markov Model (HMM), they related these features to hidden variables. Then, a Bayesian multiple change model used these variables as attack indicators. To evaluate the proposed approach, a SIP simulator was developed to generate normal messages and the Nova-VSpy tool was used to generate malicious messages. One benefit of extracting 41 features and calculating ten model parameters is the high detection accuracy over many DDoS attacks with different traffic intensities; nevertheless, high consumption of memory and CPU resources is a major drawback.

Recently, Nazih et al. [22] used an *n*-gram technique and a fast linear SVM classifier to detect INVITE flooding, malformed messages, and SPIT attacks. They used a moving window of four characters over the SIP message to extract all *n*-gram tokens and then store occurrences for each *n*-gram in the features vector. An *l*1 regularizer that produces sparse solutions was used with SVM in its primal form. Two different datasets used to evaluate the proposed classifier achieved a high detection rate with a low detection time. In addition, *l*1-SVM outperformed traditional dual form SVM in detection and training times.

Tsiatsikas et al. [23] built a parser based on the Session Description Protocol (SDP) to detect malformed messages attacks exploiting the SIP message body. To make sure that the SIP message’s body is correct according to SDP syntax, the parser was fitted with 100 different rules. High accuracy was achieved with little processing time overhead, but the parser rules are dedicated only to the SDP part of the SIP message.

The previously mentioned related work used statistics-, rules-, or finite state machine-based approaches or traditional machine learning approaches such as SVM. Rozhon et al. [24] considered the exchange of signaling messages in VoIP networks as a simple language and used RNN to create a model that detects the changes in message sequences as malicious behavior. They considered each SIP dialog as a separate sequence and used one-hot-vector to extract its features. The proposed approach was tested using a small dataset and achieving a detection accuracy of 82–96%. To the best of our search, deep learning approaches such as RNN are not used to detect DDoS attacks on VoIP networks.

## 3. Proposed Approach

The principal objective of the proposed approach is to use deep learning techniques to develop and train a model that can learn the features of SIP messages automatically to detect DDoS attack patterns in an efficient and timely manner. This model was built in two phases, the first to extract features from SIP messages and the second to use an RNN model to detect DDoS attacks.

### 3.1. Feature Extraction

**Figure 1:**
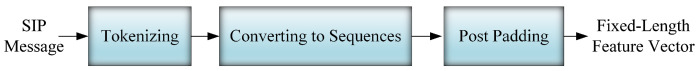
Feature extraction process.

In contrast to previous machine learning approaches to DDoS detection such as those in [9,10], our method does not require designing features that represent SIP messages, as show in Figure 1. The feature extraction process consists of tokenizing, converting to sequences, padding, and embedding. All punctuation is removed in the tokenizing step, in which the SIP message is converted to a list of tokens separated by spaces. In the second phase, every token is converted to its index in the dictionary that was created in the previous phase. In the third phase, we use post padding in which we add zeros at the end of every feature vector to convert all feature vectors into fixed-length vectors.

Although bag-of-words [25] representation is frequently used in text processing tasks, recently word embedding [26] has been found to provide better performance as it extracts semantic and syntactic features of the text and captures similarities between words. In our problem, we found that SIP messages usually do not have many English language words. Therefore, we do not use a pre-trained word embedding method such as Global Vectors (GloVe) [27] in the last phase of our feature extraction process. As an alternative to word embedding, a TensorFlow [28] token embedding layer is used to map vectors of tokens from discrete to continuous representation. This layer has the additional advantage of weights updating during backpropagation, which might enhance the proposed approach’s performance.

### 3.2. RNN Model

An RNN [29] is a neural network designed for sequential data that is widely used in many fields such as natural language processing [13].

**Figure 2:**
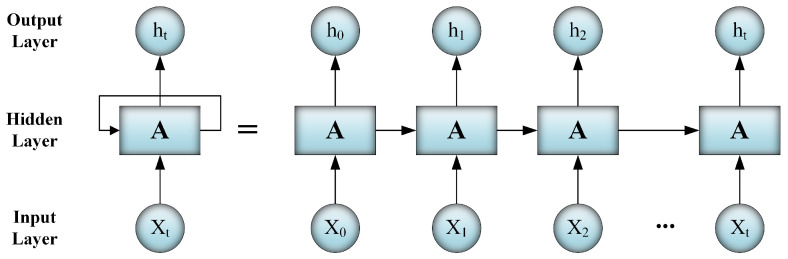
The architecture of an RNN and its unrolled version.

The main difference between an RNN and a traditional feed-forward neural network is the feedback loops of the hidden units. An RNN learns through previous time steps; in the hidden layer, the output of each node in the previous time step is considered as an input to the same node in the current time step. The node’s memory stores the required information to be used for learning in future time steps. The architecture of an RNN is shown in the left part of Figure 2, while the right part has an unrolled RNN.

Bidirectional Recurrent Neural Networks (BRNNs) were introduced in [30] to overcome the constraints of RNNs by connecting two hidden layers to run in opposite directions. This allows these layers to receive information of previous and next states. BRNNs have been found to be more effective than RNNs for some problems such as speech recognition [31] and phoneme classification [32].

Similarly to other neural network architectures, RNNs suffer from the vanishing gradient problem as a result of the backpropagation algorithm used in training, which causes RNNs to be limited to deal with only short sequences. Many RNN variants, such as Long Short-Term Memory (LSTM) [33] and Gated Recurrent Unit (GRU) [34], have been proposed to handle the long-term dependency problem.

LSTM uses a gating mechanism to optimize information passing through. First, a sigmoid function layer outputs either one or zero. This functions passes all information in the case of one, and no information in the case of zero. Each LSTM unit contains three gates, forget, input, and output. The forget gate removes outdated memory, the input gate obtains new data, and the output gate combines short-term and long-term memory to create the current memory state. The forward updates at each time step *t* are as follows:

(1) $$\begin{array}{cc} \; & i_{t}=\sigma \left(W_{i}h_{t-1}+U_{i}x_{t}\right) \end{array}$$

(2) $$\begin{array}{cc} \; & f_{t}=\sigma \left(W_{f}h_{t-1}+U_{f}x_{t}\right) \end{array}$$

(3) $$\begin{array}{cc} \; & o_{t}=\sigma \left(W_{o}h_{t-1}+U_{o}x_{t}\right) \end{array}$$

(4) $$\begin{array}{cc} \; & c_{t}=f_{t}⊙c_{t-1}+i_{t}⊙\tanh\left(W_{c}h_{t-1}+U_{c}x_{t}\right) \end{array}$$

(5) $$\begin{array}{cc} \; & h_{t}=o_{t}⊙\tanh\left(c_{t}\right) \end{array}$$

where $i_{t}$ is the input gate, $f_{t}$ is the forget gate, $o_{t}$ is the output gate, $c_{t}$ is the memory cell, and $h_{t}$ is the hidden state. ⊙ denotes element-wise multiplication. $W_{i}$, $U_{i}$, $W_{f}$, $U_{f}$, $W_{o}$, $U_{o}$, $W_{c}$, $U_{c}$, are the parameters of the LSTM network.

A GRU has a simpler architecture since it combines the forget and input gates into a single update gate. It is a simpler version of LSTM that can be trained more rapidly owing to its having fewer parameters. Its forward updates at each time step *t* are as follows:

(6) $$\begin{array}{cc} \; & z_{t}=\sigma \left(W_{z}h_{t-1}+U_{z}x_{t}\right) \end{array}$$

(7) $$\begin{array}{cc} \; & r_{t}=\sigma \left(W_{r}h_{t-1}+U_{r}x_{t}\right) \end{array}$$

(8) $$\begin{array}{cc} \; & {\tilde{h}}_{t}=\tanh\left(W_{h}\left(h_{t}⊙r_{t}\right)+U_{h}x_{t}\right) \end{array}$$

(9) $$\begin{array}{cc} \; & h_{t}=z_{t}⊙h_{t-1}+\left(1-z_{t}\right)⊙{\tilde{h}}_{t} \end{array}$$

where $z_{t}$ is the update gate, $r_{t}$ is the reset gates. $W_{z}$, $U_{z}$, $W_{r}$, $U_{r}$, $W_{h}$, $U_{h}$, are the parameters of the GRU network.

**Figure 3:**
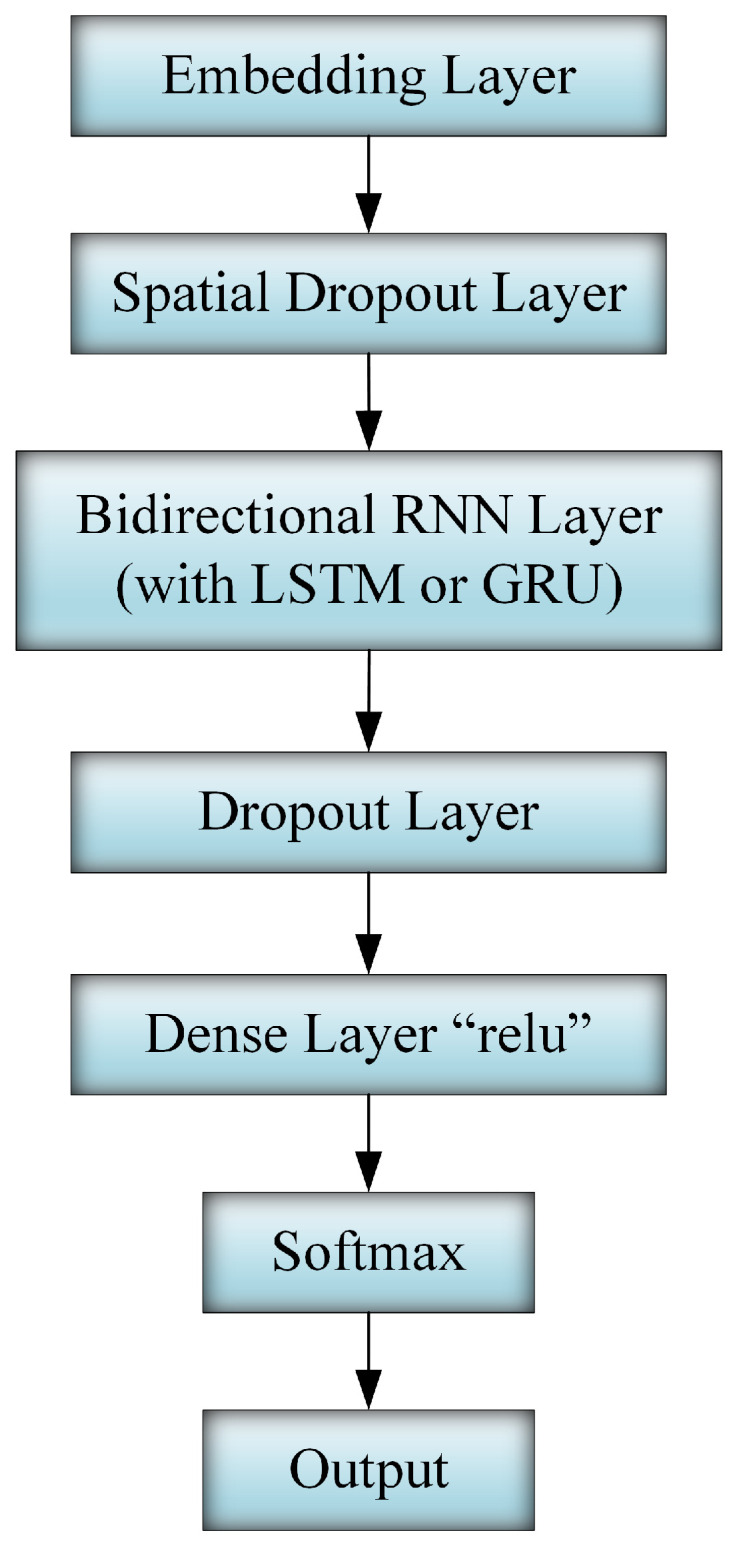
Full deep neural network model.

A GRU has a few numbers of parameter matrices, but LSTM achieves better accuracy with long sequences. In our proposed approach, we used a BRNN and leveraged LSTM and GRU, as shown in Figure 3.

Since deep learning neural networks are susceptible to overfitting the training dataset, we randomly drop the outputs of some layers out during training [35]. This dropout is an effective regularization method that reduces the probability of overfitting and enhances the generalization of the unseen dataset.

A Rectified Linear Unit (ReLU) [36] was used as the hidden layer activation function. This is a piecewise linear function that outputs the same input if it is positive and outputs zero otherwise. Moreover, it is fast and facilitates the training process.

Since we formulate DDoS attack detection as a multi-class classification problem, we use the softmax activation function in the final layer of our model (i.e., output layer). This function converts numbers into probabilities with their sum equal one and outputs probability distributions of a list of possible outputs as a vector. Finally, cross-entropy was used as a loss function that calculates the sum of the average difference between the actual and predicted probability distributions for all classes.

## 4. Dataset

In VoIP, there is a serious lack of shareable traffic datasets and no benchmark dataset to assure the reliability of the proposed approach. To evaluate our proposed approach, we built our dataset using real traffic traces that were injected with malicious messages. In the next subsections, we explain the capturing of the real traffic, the generation of the malicious traffic, and the preprocessing procedure for merging them to develop well-balanced datasets.

### 4.1. Real Traffic Traces

Collecting a real SIP dataset is very important for validating our approach. Many researchers have created their synthetic datasets using a laboratory testbed. Sometimes, a shortcoming of such datasets is that the produced traffic might not represent real VoIP traffic exactly.

To build our real traffic dataset, we arranged with an educational institution to capture its VoIP traffic. The institution’s VoIP network has five servers and about 4000 VoIP phones distributed over the main campus and four branches in different geographical places. After obtaining the required approvals, the network administrator ran a capture tool to save the VoIP traffic randomly during working hours for two months. This dataset contains many types of SIP messages, below is a sample of a real REGISTER message.
REGISTER sip:187.154.89.18 SIP/2.0Via: SIP/2.0/TCP 187.22.29.58:63968;branch=z9hbK74e9eab8From: <sip:9787@187.154.89.18>;tag=b07d4770d11731e408d-090de6b4To: <sip:9787@187.154.89.18>Call-ID: b07d47d0-f9e70003-190fa99c-78ae4774@187.22.29.58Max-Forwards: 70Session-ID: db4ad297105a000b07d47d0f9e7CSeq: 3431 REGISTERUser-Agent: CUCMContact: <sip:13dffdb7-13f3-41ba-93d7-d8eb29b71@187.22.29.58:63968;transport=tcp>Content-Length: 0Expires: 3600

### 4.2. Malicious Messages

The most common DoS attacks are malformed messages and flooding. In the malformed messages attacks, the attacker sends a modified version of a correct SIP message, which in turn causes a partial failure or a restart of the SIP device when attempting to process this message. Flooding attacks generate a large number of SIP messages to force the SIP device to consume resources such as memory. Therefore, the SIP device is going to be out of service for legitimate users [37].

To launch the flooding attack, the attacker may use INVITE, REGISTER, and BYE methods. Still the most common and critical among them is the INVITE flooding as it leads to the exhaustion of the target’s resources (i.e., bandwidth, CPU, and memory) by sending a large number of INVITE messages. The attacker could be a legitimate user if such attacker has an account in the SIP server, or even an intruder who has violated the authentication requirements. Furthermore, this attack can be launched using a single source (i.e., DoS attack) or multiple sources (i.e., DDoS attack) to send INVITE messages to the end-user or the server [38].

The previous real traffic traces contain only benign messages and no malicious SIP messages. The SIPp-DD [39] is a command-line tool used to generate the required INVITE flooding DDoS messages. The most important advantage of SIPp-DD is the spoofing mechanism implementation to support the distributed attacks generation. In addition, the attack scenario can be defined using an Extensible Markup Language (XML) script which contains the structure of the SIP message that will be generated. Furthermore, Comma Separated Values (CSV) files can be used to insert values into any part of the SIP message during their creation in the attack. Below is a sample of an INVITE message that was generated by this tool.
INVITE sip:9322@24.203.127.58:5060;transport=tcp SIP/2.0Via: SIP/2.0/TCP 24.203.127.58:5060;branch=z9hG4bK-35415-0From: "user" <sip:9527@24.203.127.58>;tag=b07d47d0d3c283c9c20-2e0600c4To: <sip:9322@24.203.127.58>Call-ID: b047d0-e05d8b-0789cb-5bad2e@24.203.127.58User-Agent: CP8861/11.7.1CSeq: 101 INVITEExpires: 180Contact: <sip:1176@24.203.127.58:5060;transport=tcp>Max-Forwards: 70Content-Type: application/sdpContent-Length: 204
v=0o=CCM-SIP 9727210 1 IN IP4 24.203.127.58s=SIP Callc=IN IP4 157.208.158.171t=0 0m=audio 6000 RTP/AVP 8 101a=rtpmap:8 PCMA/8000a=rtpmap:101 telephone-event/8000a=rtpmap:101 0-15

To cover most attacks possibilities, we created three scenarios with different attack durations and different attack intensities, similarly to previous work in [9,17]. The attack durations of these scenarios include both small (30 s) and long durations (60 and 120 s). Moreover, for every scenario we have five attack intensities; very high (VH), high (H), medium (M), low (L), and very low (VL), meaning flooding the VoIP network with 500, 100, 50, 25, and ten messages/s, respectively.

### 4.3. Data Preprocessing

Malicious messages are conceptually simulating benign messages. Using datasets with large differences between benign and malicious messages leads to unrealistic high performance [40]. To avoid this in our experiments, our attack simulation tool generates traffic that mimics the real traffic.

To achieve this in the malicious traffic, we did not add any remarkable SIP headers such as “Subject: Performance Test” in the developed SIPp-DD scripts and used the available information regarding the educational institution such as users’ extension and contacts’ names in the generation process using CSV files.

For the benign traffic, we developed a tool that performed the next preprocessing steps, extracting only the SIP traffic, removing the SIP optional headers that contain the hardware manufacturer-specific information such as “Manufacturer-Guide,” and replacing the Arabic contact names with the equivalent English names.

Finally, the tool injected DDoS attack messages in the real traffic traces maintaining an equal ratio of benign and malicious messages to evade imbalance between the dataset classes. In addition, it divided each scenario’s dataset randomly into training (60%), validation (20%), and testing datasets (20%).

The final output is four datasets, one for every developed scenario with six message classes and with the last being the merging of the three datasets with 16 message classes to be used in creating an RNN model for deployment. Table 1 summarizes the datasets’ details.

## 5. Experiments

Our proposed approach used the RNN model and reported its performance when associated with LSTM and GRU. In addition, it was compared with a classical machine learning approach that utilized *n*-grams for feature extraction and SVM in its primal form and *l*1 regularization (i.e., linear *l*1-SVM) [22] in terms of detection accuracy and detection time per SIP message.

All of the experiments were implemented using Python. The deep neural networks were implemented using TensorFlow [28] on a Tesla (R) P100 GPU, and the classical machine learning approach was implemented using Scikit-learn [41] and Intel(R) Xeon(R) CPU 2.2 GHz. The available memory was 24 GB and the hardware was available at no cost through Google Colab.

### 5.1. Setup and Evaluation

The RNN model’s hyper-parameters have a substantial effect on system performance. The performance might be improving or worsening based on the values of these hyper-parameters. At the beginning of our experiments, we conducted numerous discovery experiments and attempted different model structures such as RNN, BRNN, and different numbers of hidden layers. We have found that the proposed structure shown in Figure 3 has given the best results over our dataset. In addition, nearly all of the model hyper-parameters were attempted before settling on the following values: the embedding layer dimension, 50; the number of units in the hidden layer, 16; the dropout, 0.2; the learning rate, 0.001; and the number of training epochs, 100.

At the beginning of our experiments, we conducted numerous discovery experiments and attempted different model structures such as RNN, BRNN, and different numbers of hidden layers. In addition, nearly all of the model hyper-parameters were attempted before settling on the model architecture illustrated in Figure 3 associated with the following values: the embedding layer dimension, 50; the number of units in the hidden layer, 16; the dropout, 0.2; the learning rate, 0.001; and the number of training epochs, 100.

**Figure 4:**
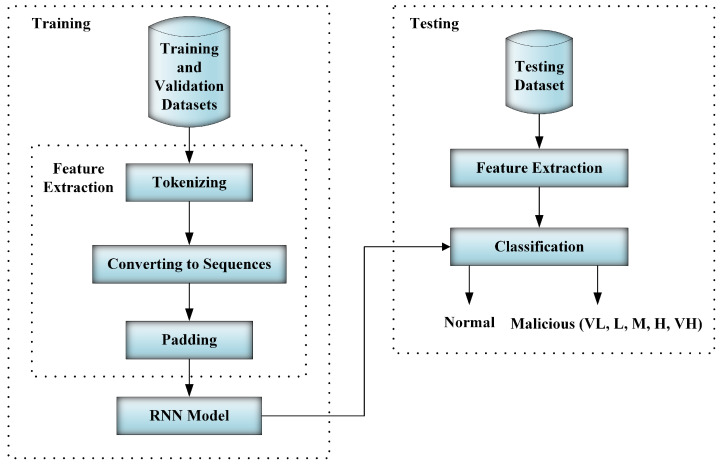
Training and testing workflow.

Deep learning models are typically prone to overfitting. The validation dataset was used to assist in preventing overfitting. In training, the training dataset was used for model training and the validation dataset was used to evaluate model performance. Later, the testing dataset, considered to be unseen data for the model, was used to evaluate the model and report the testing results as shown in Figure 4.

The F1 score was used for assessing the performance of our approach. It is the harmonic average of the precision and recall that takes into account the false positives and false negatives [42]. The precision measures the correctly predicted positive instances from all the predicted positive instances, while the recall measures the correctly predicted positive instances from all the actual positive instances. The F1 score’s value is between zero and one. The higher the F1 Score, the better is the model’s performance.

(10) $$\mathrm{Precision}=\frac{\mathrm{True}\;\mathrm{Positive}}{\mathrm{True}\;\mathrm{Positive}\;+\;\mathrm{False}\;\mathrm{Positive}}$$

(11) $$\mathrm{Recall}=\frac{\mathrm{True}\;\mathrm{Positive}}{\mathrm{True}\;\mathrm{Positive}\;+\;\mathrm{False}\;\mathrm{Negative}}$$

(12) $$F1=2*\frac{\mathrm{Precision}\;*\;\mathrm{Recall}}{\mathrm{Precision}\;+\;\mathrm{Recall}}$$

It is worth to be mentioned that the accuracy quantifies all correctly classified instances (true positives and true negatives) and is given by:

(13) $$\mathrm{Accuracy}=\frac{\mathrm{True}\;\mathrm{Positive}\;+\;\mathrm{True}\;\mathrm{Negative}}{\mathrm{True}\;\mathrm{Positive}\;+\;\mathrm{False}\;\mathrm{Positive}\;+\;\mathrm{True}\;\mathrm{Negative}\;+\;\mathrm{False}\;\mathrm{Negative}}$$

The F1 score is usually more powerful than accuracy in the case of different classes’ distribution [43]. As a matter of fact, DDoS attacks have different attack intensities. Hence, the F1 score will be more effective in measuring the performance of any classifier of DDoS attacks. Moreover, it is well known (as can be seen from the definitions) that if true positives and true negatives are more vital then accuracy is the choice. On the other hand, the F1 score is used when the false negatives and false positives are critical. In fraud detection, the cost associated with false negative or false positive will be extremely high. In this case, the F1 score will be the best choice to quantify a classifier.

In each experiment, we calculated F1 score of the training and validation datasets, loss of the training and validation datasets, total training time, and average detection time per message. In addition, we plotted the confusion matrix to detect misclassified classes. If the classifier fails to assign the correct labels for the messages of a certain class, we consider it a misclassified class.

### 5.2. Results and Discussion

The character was selected as the primary unit of the feature extraction process in the first experiment. Character-based features might be slow in training and attack detection when compared with token-based features, but it does not have the Out-of-Vocabulary (OOV) problem because its dictionary contains all language characters, numbers, and punctuation marks. For the classification process, we used LSTM and GRU as well as the RNN model and reported their performance. The last classifier, *l*1-SVM, was used as a classical machine learning classifier for comparison with the proposed deep learning classifiers.

Table 2 shows the results of this experiment over the developed scenarios. RNN-LSTM outperformed other classifiers but failed to detect low-rate attacks (i.e., L and VL attacks) in the last scenario. RNN-GRU and *l*1-SVM appear to have failed to cope with long sequences when using character-based features and therefore showed a noticeably lower detection rate.

Extracting character-based features in the earlier experiment failed to discover low-rate attacks correctly. Therefore, we used feature extraction depending on tokens, not characters, in the next experiment. To minimize the OOV problem, we used a large dictionary (1000 tokens). Table 3 shows that RNN-LSTM and RNN-GRU achieved a comparably high detection rate, while the *l*1-SVM failed to discover the low-rate attacks (i.e., L and VL attacks).

**Figure 5:**
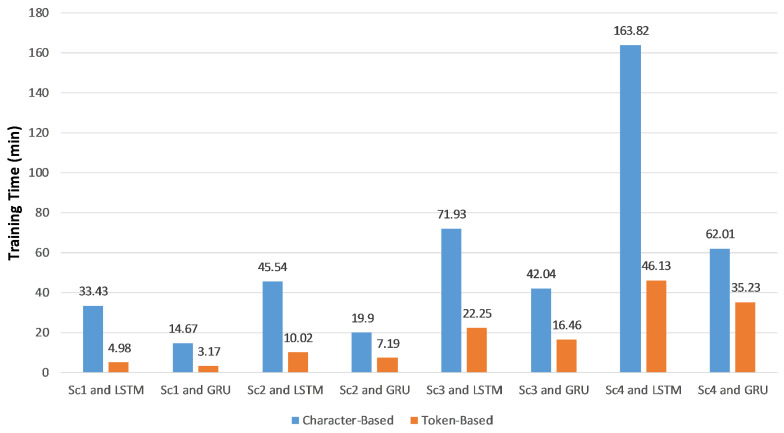
Training times of the proposed approach on a GPU system using character-based and token-based feature extraction.

**Figure 6:**
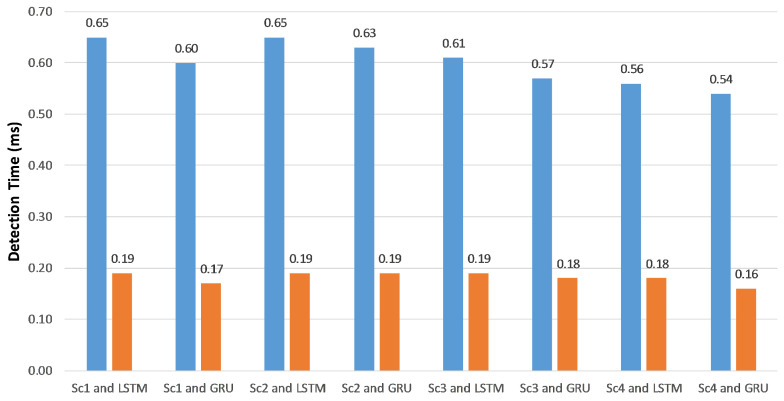
Detection times of the proposed approach on a GPU system using character-based and token-based feature extraction.

Measuring training and detection times is a significant metric in our experiments. Figure 5 shows the total training time in minutes, which equals the feature extraction time plus the classifier training time regarding the training and validation datasets. The average detection time per SIP message in milliseconds, which calculated over the testing dataset is displayed in Figure 6.

Using token-based features reduced the training and detection times because the average length of feature vectors was less than that of character-based feature vectors. Consequently, less processing power and memory were used. According to Figure 6, the detection time was about the third of the character-based detection time. In both the character- and token-based experiments, the detection times of RNN-LSTM and RNN-GRU are almost the same, but the training time of RNN-GRU is typically less than RNN-LSTM.

**Figure 7:**
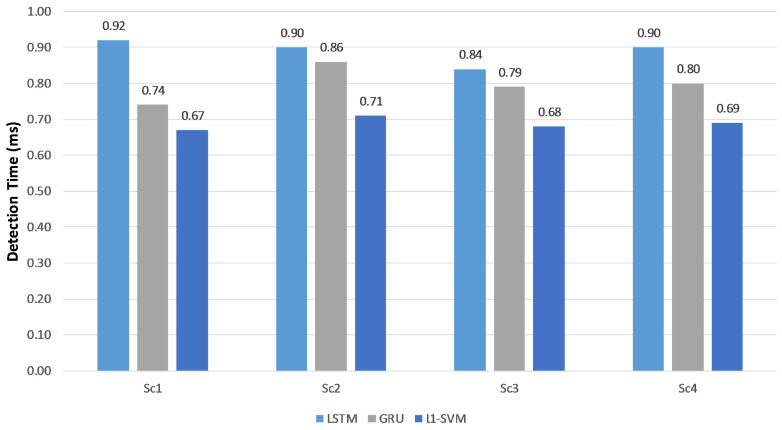
Detection time of the proposed approach on a CPU system (token-based feature extraction).

One of the most beneficial advantages of deep learning neural networks is the use of a high-speed processor (GPU). These are not readily available for classical machine learning approaches such as an *l*1-SVM. In addition, GPU is special hardware that might not be available in most deployed systems. To compare all examined classifiers, we ran the previous experiment in addition to the *l*1-SVM classifier over a CPU system and report the detection time in Figure 7. *l*1-SVM was the fastest detection classifier but still had the issue of low-rate attacks detection.

Form the previous experiments, it can be noted that using token-based feature extraction achieves a better detection accuracy than char-based feature extraction. Moreover, RNN-LSTM and RNN-GRU achieve a comparable high detection accuracy, meanwhile, they outperform *l*1-SVM. While *l*1-SVM achieves the lowest detection time on CPU, it can not detect low-rate attacks.

## 6. Conclusions

Deep learning is considered as state-of-the-art in many fields, such as intrusion detection systems. We proposed a new approach to the detection of DDoS attacks on VoIP networks. This approach used token-based feature extraction and an RNN model. Different RNN architectures were tested and the performance was compared to determine the best model architecture for the DDoS problem. High detection accuracy and low detection time were achieved over a real VoIP dataset with various attack scenarios. Given that RNN-GRU has a simpler architecture and a few numbers of parameter matrices, it achieves low detection and less training times than RNN-LSTM. Furthermore, it outperformed the classical machine learning approach *l*1-SVM. In our estimation, the detection speed it achieved qualifies our approach for online attack detection in networks. We believe that RNN can detect DDoS attacks. However, due to recurrent connections, RNN is not very efficient for parallel processing. To overcome this problem, we plan to use Convolutional Neural Networks (CNNs) or Self Attention Networks (SANs) in future work. In addition, we intend to test the proposed approach in a real VoIP network and attempt to reduce the detection time on CPU systems. 

## Figures and Tables

**Table 1:** The details of the datasets.

Scenario	Duration (s)	Number of Messages	Message Classes
Sc1	30	44,247	Normal, VH, H, M, L, and VL
Sc2	60	85,709	Normal, VH, H, M, L, and VL
Sc3	120	170,559	Normal, VH, H, M, L, and VL
Sc4	30, 60, and 120	300,515	Normal, Sc1:VH, Sc1:H, Sc1:M, …, Sc3:L, Sc3:VL

**Table 2:** Detection accuracy of the proposed approach (character-based feature extraction).

Scenario	Classifier	F1 Score	Misclassified Classes
Sc1	RNN-LSTM	99.9%	nothing
Sc1	RNN-GRU	87.45%	H, M, L, and VL
Sc1	*l*1-SVM	87.46%	H, M, L, and VL
Sc2	RNN-LSTM	99.9%	nothing
Sc2	RNN-GRU	87.04%	H, M, L, and VL
Sc2	*l*1-SVM	86.9%	H, M, L, and VL
Sc3	RNN-LSTM	100%	nothing
Sc3	RNN-GRU	86.98%	H, M, L, and VL
Sc3	*l*1-SVM	86.94%	H, M, L, and VL
Sc4	RNN-LSTM	99.36%	Sc1: M, L, and VL - Sc3: VL
Sc4	RNN-GRU	68.53%	Almost all attack classes
Sc4	*l*1-SVM	72.46%	Almost all attack classes

**Table 3:** Detection accuracy of the proposed approach (token-based feature extraction).

Scenario	Classifier	F1 Score	Misclassified Classes
Sc1	RNN-LSTM	100%	nothing
Sc1	RNN-GRU	100%	nothing
Sc1	*l*1-SVM	97.6%	L and VL
Sc2	RNN-LSTM	99.9%	nothing
Sc2	RNN-GRU	99.9%	nothing
Sc2	*l*1-SVM	97.5%	L and VL
Sc3	RNN-LSTM	99.9%	nothing
Sc3	RNN-GRU	100%	nothing
Sc3	*l*1-SVM	98.23%	L
Sc4	RNN-LSTM	100%	nothing
Sc4	RNN-GRU	99.9%	nothing
Sc4	*l*1-SVM	97.42%	Sc1: M, L, and VL - Sc2: L and VL - Sc3: L
